# Supplementary material for: An extended and generalized framework for the calculation of metabolic intervention strategies based on minimal cut sets
Source: PLoS Comput Biol. 2020 Jul 27;16(7):e1008110. doi: 10.1371/journal.pcbi.1008110 (PMC7410339; doi:10.1371/journal.pcbi.1008110)
Supplement: Attachment — Submitted filename: Review_Response_final.docx [file pcbi.1008110.s006.docx]

**Response to the comments of the reviewers**

We thank the reviewers for the careful evaluation of our manuscript and the constructive comments. Below we give a detailed response to all suggestions and questions of the reviewers and describe the respective changes in the manuscript (changes are also highlighted in the revised manuscript file).

**Reviewer #1:**

The authors extend the already existing CellNetAnalyzer for the computation of MCSs in metabolic networks. The two major additions are the integration of constraints allowing to describe contradicting conditions of the network, e. g. aerobic and anaerobic growth, and compression rules which reduce the size of the network, while including gene rules and therefore not only reactions. They show the improvements of their method by applying it to the core and genome-scale version of E. Coli. Their computational time is significantly reduced and they were able to compute MCSs in the genome scale network which was not possible before.

Overall impression:

I think the authors made a considerate improvement for the computation of MCSs in genome-scale metabolic networks. However, I think further tasks have to be executed to fully show the general applicability of their method. I am especially concerned about their claim to be able to add new reactions to their metabolic network which they do in fact “by hand”, thus this feature is not provided by their method but is part of the input which is created by the user.

**Major:**

**Reviewer 1 comment 1:** Extension 3: Where does the additional reactions come from? I do not really see a “proof of concept” for the addition of reactions. Adding new reactions to the network “by hand” is changing the input, thus this is not part of the introduced method or a new “computational” addition. I would rather like to see an automated way, similar to how it was done here <https://doi.org/10.1186/1752-0509-6-30> where the KEGG-data base was used.

**Author response:** The reviewer is right that several methods have already been published that address the addition of (a minimal set of) reactions from a repository in order to enhance the model with certain phenotypes. This includes the SMILEY algorithm used in the reference cited by the reviewer. However, our presented extended MCS algorithm is more general in this regard as it finds combinations of both reaction additions AND deletions in order to solve the intervention problem. This requires a more complex duality-based MILP for MCS calculation and leads to an enormous increase of the search space compared to the pure reaction addition problem (the latter can be addressed entirely in the primal system as in SMILEY). As input for an MCS problem allowing both reaction additions and deletions one needs to define (i) the given network with the set of deletable reactions and non-targetable reactions and (ii) a repository of reactions that can be added. The original (given) network is augmented with the reaction addition candidates from the repository and these reactions are then marked as “addable”. In principle, for the repository of reactions we could indeed use any reaction database (e.g. KEGG) as used in the reference cited by the reviewer, however, together with the deletable reactions from the given network, this would quickly inflate the search space to a size that cannot be treated by the solver. In our application, we therefore did the following: the heterologous 2,3-BDO pathway was already included in the provided E. coli network (thus, not part of the repository anymore) whereas the repository of addable reactions consisted of the different substrates (i.e. their uptake reactions). The algorithm searches for (cost-)minimal combinations of substrate(s) (including co-feeding) and suitable knockouts. The resulting optimal substrate (co-feeding) + knockout strategies are thus delivered by the solver and not designed manually. Therefore, this case presents an example for truly solution-dependent reaction additions. Surely, the repository of addable reactions was not very large in this example, however, the possibility to search for certain combinations of substrates increases the search space tremendously, typically much more than adding an intracellular reaction. Hence, in our application the goal was not to find the optimal 2,3-BDO pathway from a reaction repository but to search for minimal substrate combinations that might be beneficial in conjunction with reaction knockouts in order to induce growth-coupled product synthesis along the already inserted heterologous pathway. Nevertheless, our MCS approach could also use repositories that include, for example, different variants of product synthesis pathways and then identify the one that allows optimal induction of growth coupling (in combination with suitable knockouts). However, the size of the repository that can be handled by the algorithm will always depend on dimension and structure of the problem and some preselection of reaction addition candidates (from large databases such as KEGG) will often be necessary to obtain smaller repositories of addable reactions. We also note that our extended MCS approach could, in principle, be used to mimic SMILEY, i.e., to search exclusively for reaction additions that enable a certain phenotype (but this is not the targeted main application and might better be addressed by dedicated methods such as SMILEY).

We address the combinatorial complexity of reaction additions in the MCS framework as well as the availability of other methods searching for reactions additions in the Discussion section:

*“As another major extension, we generalized the MCS framework to now also allow combinations of reaction/gene deletions and additions and to assign arbitrary cost factors to each intervention. We exemplified this feature for finding substrate co-feeding strategies for growth-coupled strain designs, however, other applications include the search for strain designs where, in combination with suitable gene/reaction knockouts, heterologous reactions or pathways are added to the network to achieve optimal production behavior with minimal intervention costs. Clearly, as demonstrated in the application example, offering a larger number of addable reactions may vastly increase the solution space, especially when searching for co-feeding strategies on multiple possible substrates. Hence, the repository of optional insertions should be limited to a manageable number of (preselected) reactions or pathways. Clearly, as demonstrated in the application example, offering a larger number of addable reactions may vastly increase the solution space, especially when searching for co-feeding strategies on multiple possible substrates. Hence, the repository of optional insertions should be limited to a manageable number of (preselected) reactions or pathways. …. We note that some of the presented features, in particular substrate co-feeding or reaction additions, have partly been addressed also in previous studies that used bi-level optimization approaches [7,8,33,34,63]. Yet, neither individual cost factors nor reaction additions and co-feeding strategies have so far been used in the more general framework of MCS. Moreover, co-feeding strategies, where an external metabolite may act as (addable) substrate or byproduct, requires the definition of multiple target regions and can thus only be handled by the extended MCS approach.”*

**Reviewer 1 comment 2:** I would like to see a wider application, not only for the two E. Coli models. Maybe the authors can apply their method to all metabolic networks from the BiGG model data base, in order to see what the benchmarks are and the limits (regarding the sizes of the networks) of their method. Right now they claim to introduce a new toolbox, but do not show the overall applicability of it.

**Author response:** We agree that the applicability, performance, and potential limits of the new approach could be shown by computing MCS in other (including larger) models. We followed this suggestion to extend our exemplary calculations for two additional genome-scale networks (see below). However, for the following reasons, we did (or could) not redo similar calculations for all models listed in the BiGG database:

1) First of all, iML1515 is actually a quite large model when compared to other models in the BIGG database. Many of the relatively few larger models in BiGG are unsuitable production hosts and thus not meaningful for strain design calculations (e.g. *Homo sapiens* or *Cricetulus griseus*). Furthermore, 58 of the 101 BIGG models are also *E. coli* models, that are partly outdated (e.g. iAF1260 and iJO1366 have been replaced by iML1515) or have either similar or even smaller size. Hence, most BIGG models would not be relevant for further comparative calculations in our paper.
2) Concerning the automated setup of MCS calculations in models of the BiGG database (which would be necessary to follow the suggestion of the reviewer), there are several technical particularities among the models that hamper the construction of a one-fits-all pipeline, e.g. for computing 2,3-BDO strain designs. To only mention a few: (different) parts of the 2,3-BDO must be added to the respective models, depending on which metabolites/reactions of the pathway are already present. For the computation with two desired regions, the ATP maintenance reaction must be present (e.g. not the case in iNJ661 or iAM_Pb448) and not be fixed (e.g. iYO844). Extreme boundaries like +/-999999 must be adapted to avoid numerical issues from variable values spanning too many orders of magnitude in the MILP. Hence, almost every model will require a careful pre-analysis and manual adaptations before it can be used in computations such as shown in Tables 1 and 2. We feel that this huge effort would not be reasonable.
3) We certainly do not want to convey the impression that the presented MCS algorithm has no limitations in network size or problem complexity. For example, a complete enumeration of MCS up to size 7 was done in iML1515. However, the enumeration of all MCS up to the size of 9 in iML1515 failed because of memory overflow. We now mention those limitations explicitly in the Discussion section:

*“Generally, a full enumeration of all MCS in genome-scale models is typically only feasible up to a certain maximal number of interventions (for example, an enumeration of MCS with 9 or more interventions for 2,3-BDO synthesis in iML1515 was not possible due to memory overflow). “*

Also, even if we would do more calculations with other models, it will not be possible to find a generic rule predicting up to which network/MCS size the computation of MCS would be possible, since this will heavily depend on the network structure, the type and complexity of the MCS problem as well as on the hardware or compute cluster that is available.

To nevertheless provide additional examples with alternative organisms (including a eukaryotic one) and with different model sizes, we selected two more genome-scale models. One is the yeastGEM model (available on GitHub), which is the currently best curated model for *Saccharomyces cerevisiae* and larger than iML1515, and the second network is the *Pseudomonas putida* model iJN746 from the BiGG database, sized between iML1515 and ECC2. We adapted the existing MCS computation pipeline to compute 2,3-BDO strain designs and could show consistent trends, e.g. regarding the beneficial effect of our GPR compression rules. We added a paragraph for these calculations to the Results section (as well as a Supplementary Table S5 with a list of the results and scripts and models to repeat these computations on GitHub):

*“To finally demonstrate that our extended MCS approach also works with other metabolic networks, including an eukaryotic one, we repeated all calculations for growth-coupled 2,3-BDO synthesis with E. coli (Tables 1 and 2) in the genome-scale networks of Saccharomyces cerevisiae (yeastGEM [61]; 1147 genes and 3989 reactions) and Pseudomonas putida (iJN746 [62]; 746 genes and 1054 reactions). We found similar trends in the benchmark computations (see S5 Table). The GPR compression rules are particularly beneficial for the yeast model where we found a speedup factor of 20, while it is 2.2 for the smaller network of P. putida. The scripts to reproduce these scenarios are also available under the GitHub link.”*

Furthermore, please note that we also added another benchmark calculation related to synthetic lethals as suggested by Reviewer 2 (see below).

**Reviewer 1 comment 3:** For me, the major achievement here is not that their method is faster, but that they were able to compute MCSs in the genome-scale E. Coli which was not possible before. They should emphasize this more. Also, by applying it to other genome-scale networks and show where they are able to compute MCSs and the non-decompressed version not.

**Author response:** Actually, the ability to compute MCS in genome-scale networks (via the dual approach) is not new and was demonstrated by us for the first time in 2014 (<https://doi.org/10.1371/journal.pcbi.1003378>) and was then later used by us and others (e.g., <https://www.nature.com/articles/ncomms15956>; <https://www.nature.com/articles/s41467-017-00555-y>). We think this needs no special mentioning or demonstration anymore. However, the major new features for the MCS framework added in this work are (1) the specification of multiple target and desired regions; (2) the possibility to consider combinations of reaction deletions and additions; (3) the computation of substrate co-feeding strategies; (4) an improved version for the integration of gene-protein-reaction (GPR) associations into the metabolic model and a number of effective preprocessing and compression steps to reduce the GPR rules and thus to accelerate the computation of gene MCS; and (5) the possibility to associate individual cost factors to each intervention. As mentioned, some of these features have partly been addressed also in previous studies that used bi-level optimization approaches (e.g., inclusion of GPR rules in strain design), however, none of these features were so far available in the more general MCS framework and some feature are certainly unique (e.g., GPR rule compression, consideration of multiple target and desired regions).

Maybe the reviewer was confused by the different network compression routines: network compression has been used also in previous studies (e.g. <https://doi.org/10.1371/journal.pcbi.1003378>) and was also there essential for dealing with genome-scale networks. However, these studies did only consider reaction knockouts, but no GPR rules. Here we integrated GPR rules in the network and developed, on top of the standard network compression techniques, additional compression rules for GPR associations which further reduce the complexity of the problem considerably if GPR rules are applied (factor 4-15 faster compared to sole network compression without GPR rule compression; see Table 1). The advantage of these new GPR compression rules is that they can also be applied in conjunction with other strain optimization methods.

Regarding the application of our extended MCS framework to other networks see response to comment 2 of the same reviewer.

**Reviewer 1 comment 4:** Is the computation of MCSs using CellNetAnalyzer the same as the non-decompressed column in table1? If yes, please clarify. If not, the authors should compare their method with CellNetAnalyzer and/or other state-of-the-art MCSs methods.

**Author response**: As mentioned in the response to comment 3, so far, network compression was used also in previous studies (e.g. (<https://doi.org/10.1371/journal.pcbi.1003378>) and was there also essential for dealing with genome-scale networks. However, these studies (and also the implementation in CellNetAnalyzer) did only consider reaction knockouts, no GPR rules. Here we integrated GPR rules in the network and developed, on top of the standard network compression techniques, additional compression rules for GPR associations which further reduce the complexity of the problem considerably (compare the columns “network compression” vs. Network + GPR compression in Table 1: with additional GPR compression (on top of network compression) MCS calculation is sped up by a factor of 4-15). In the text below Table 1 we write:

*“As expected, MCS computation with combined network and GPR compression has superior per­formance: compared to the traditional pure network compression it runs about 15 times faster in the core setup and reduces the runtime of the genome-scale setup by a factor of about 4* *“*

While the E.coli genome-scale problem in Table 1 was still feasible with pure network compression and without GPR rule compression, several scenarios from Table 2 would not have been treatable in reasonable time without GPR rule compression.

**Reviewer 1 comment 5:** page 17: The authors claim that decompressing the MCSs computed in the compressed network may lead to higher number of interventions per MCS. Thus they do not compute MCSs for the original network. Can they elaborate what that means? For example is a MCS of size 1 in the original network always a MCS of size 1 in the compressed network (I think the answer is yes, but not the other way around)? It could happen that a lot of “small” MCSs in the compressed network are actually too large for wet lab applications, thus computing those does not seem to be efficient. Can they compare the size of the compressed MCSs to the size of the MCSs in the original network the compressed MCSs relate to? How do they make sure that all MCSs of desired size are found?

**Author response:** The reviewer raises an important question regarding the size of the MCS with respect to the compressed and the original network. First, the reviewer’s statement is correct; an MCS of size 1 in the original network always corresponds to an MCS of the size 1 in the compressed network, but not necessarily vice versa. However, our compression routine guarantees that every MCS found in the compressed model will always decompress to at least one MCS with the same size/cost in the original model. Generally, an MCS in the compressed model will be decompressed to all corresponding MCS in the original network. This may also include oversized / overly expensive MCS (exceeding the cost threshold) which, however, can easily be identified and then be removed during post-processing.

In practice, we address this problem by combining the compression of gene rules and of reactions with the attribution of cost factors different from 1 (this is explained in GPR compression rule 7 and, for the reactions, two paragraphs later when explaining step 4 of Figure 4). For example, for compressing gene rules, this cost association is done when multiple genes are lumped in GPR-compression step 7. As an example, consider three genes that occur in a GPR rule ((g1 AND g2) OR (g1 AND g3)). During lumping, the minimum deletion cost is determined. If all genes have the deletion cost of 1, the knock-out cost of the lumped gene is set to 1, as only g1 needs to be knocked out (otherwise, if the genes were marked as addition candidates, the addition cost of the lumped gene would be set to 2). During expansion of the compressed MCS, a knock out of the lumped gene would expand to a knockout of either g1 or a knockout of g2 and g3 (but not g1, g2 and g3). Both solutions are returned, unless for instance, g2 and g3 lead to a violation of the upper cost limit for MCS.

This procedure of lumping genes is described briefly under Extension 4, compression step 7:

*“Lump equivalent gene deletion candidates that always occur together in conjunctions (e.g.,* $g_{1}\wedge g_{2}$*) and lump gene addition candidates likewise. In some cases, another lumping can be performed inside single GPR rules if the genes do not occur in any other context. Finally, repeat the substitution also for the disjunctions of type (*$g_{1\wedge2}\vee g_{3})$*. To map the original intervention costs, each lumped gene carries the minimum intervention costs of the gene rule that was substituted. Computed MCS involving lumped genes must be expanded in a post-processing step.”*

As this phenomenon was so far not discussed sufficiently, we added a brief discussion at the end of Extension 4 to clarify that all MCS that are computed in the compressed network expand to at least one MCS of the same cost in the original network:

*“As shown in this example, decompression of lumped genes and reactions will not only result in a higher number of MCS, it may also lead to a higher number of interventions per MCS or more expensive MCS. Yet, the cost-reattribution during GPR compression step 7 ensures that any MCS found in a compressed network still expands to at least one MCS in the original network with identical costs (e.g. g_((5∧9)∨7) would have deletion costs of 2 in the compressed network). In more complex cases where MCS have multiple expansions with different costs, all MCS that exceed the MCS cost limit are discarded.”*

**Reviewer 1 comment 6:** I would rather like a different structure of the article. I would like to first read the overall results, thus the improvement of their method, and the extensive description of the rules and methods afterwards.

**Author response:** Since this is a methodological article, we would like to keep the current structure and first introduce the major new (theoretical) developments before showing its applications. We believe that it would be confusing to show and discuss the results of our application example and the advantages of the new developments before giving clear definitions and presenting what these new developments actually are. For example, when showing the results of calculations with multiple target/desired regions one needs to know what is meant with multiple regions (which are terms not so well-known to many readers) and this requires a detailed and rigorous introduction of these terms before showing and discussing their impact.

On the other hand, to support readers who like to get an overview on the Results first, we extended the summary of the main results in the Introduction section:

*“In this work, we introduce a number of extensions and generalizations for the MCS framework that improve algorithmic performance and broaden the scope of applications, especially in the context of strain design. The main extensions include (1) the specification of multiple target and desired regions; (2) the possibility to consider combinations of reaction deletions and additions; (3) the computation of substrate co-feeding strategies; (4) an improved version for the integration of gene-protein-reaction (GPR) associations into the metabolic model and a number of effective preprocessing and compression steps to reduce the GPR rules and thus to accelerate the computation of gene MCS; and (5) the possibility to associate individual cost factors to each intervention. We demonstrate the relevance and applicability of these developments by computing MCS for realistic examples of genome-scale strain designs of Escherichia coli for the growth-coupled production of 2,3-butanediol. In particular, we could identify promising new strain designs that were formerly unpredictable, e.g. based on substrate co-feeding. The example calculations also confirms considerable runtime speedups when using the introduced rules for compressing GPR associations.”*

Furthermore, in principle, readers who like to read the Results first, may initially skip the Methods section and read it after the Results section. However, as mentioned above, we feel that, for this type of paper, it is more appropriate for the reading flow to start with the Methods section.

**Minor:**

**Reviewer 1 comment 7:** Extension 2: What happens if *p_i* is set to 0? Would this imply that reaction *i* is always set to zero (or non-zero). Would you still be guaranteed with a true MCS?

**Author response:** This is a really good question. The standard MCS algorithm used so far uses a “1” for all p_i. This implies that minimization of this objective function leads to a search for support-minimal MCS (MCS with minimal cardinality, i.e. with minimal number of knock-outs). Now, with the generalized objective function, the MCS still ensure that the cost function is minimized (which still justifies the use of the term “minimal” cut set). However, if, for example, some p_i are set to 0, then it is not necessarily an MCS with minimum cardinality since some reaction knockouts may be added “for free”. In those cases, there may be pairs of MCS that differ only in the presence/absence of these cost-free reaction deletions (it would be easy to filter equivalent MCS that are supersets of others due to p_i being zero; but in some case one might want to find all these alternatives). However, it is not true that a reaction with p_i=0 will always be removed: the desired region (to be protected) may require the presence of a reaction with p_i=0 and it can then not be deleted.

To clarify this point, we added the following paragraph in the “Extension 2” section:

*“There is one important change in the interpretation of MCS when using the generalized objective function (14). Minimization of the original (simpler) objective function (10) (which is equivalent to set all* $p_{i}$ *to unity in (14)) always delivers MCS with a minimum number of knock-outs, hence, of MCS with minimal support. With the generalized objective function (14), the MCS still ensure that the cost function is minimized (which still justifies the use of the term “minimal” cut set). However, if, for example, some* $p_{i}$ *are set to 0, then MCS may be found where the number of reaction knockouts is not minimal since some deletions may be added “for free”. In those cases, there can be groups of MCS that differ only in the presence/absence of these cost-free reaction deletions. It is easy to filter out equivalent MCS that are supersets of others in a postprocessing step, however, in some cases it might just be the goal to find all these alternatives. Generally, even if the* $p_{i}$ *of a reaction is zero, it might not be removable as the desired region may require the presence of this reaction.”*

**Reviewer 1 comment 8:** What does CSOM stand for? Abbreviation is not introduced (p. 3, paragraph 2)

**Author response:** Thanks, this was corrected.

**Reviewer 1 comment 9:** No introduction of “flux vector” (steady state or not? However, it is defined later) (p. 3, paragraph 2).

**Author response:** Thanks, corrected to “steady-state flux vector”.

**Reviewer 1 comment 10:** cite [https://doi.org/10.1093/bioinformatics/bty1027 on p3-4](https://doi.org/10.1093/bioinformatics/bty1027%20on%20p3-4)

**Author response:** Thanks, the reference was added.

**Reviewer 1 comment 11:** steady state vs. steady**-**state (inconsistent spelling)

**Author response:** Thanks, at one place it was indeed incorrect and corrected. The use of “steady-state” vs. “steady state” depends on whether it serves as attribute (e.g., steady-state flux vector) or as object (e.g., “… the steady state is reached …”).

**Reviewer 1 comment 12:** p5: MCS abbreviation was already introduced

**Author response:** Thanks, corrected.

**Reviewer 1 comment 13:** Formula (5): What is *Y*?

**Author response:** Thanks, corrected.

**Reviewer 1 comment 14:** what is *P* and *S* on page 7? Is it O2 up or just some general substrate product? What is *X*? What is *mu*?

**Author response:** Thanks, we now mention that P and S are a generic product or substrate. We replaced X and mu by “BM” and r_BM, respectively, to be more intuitive and consistent with the other examples.

**Reviewer 1 comment 15:** in Formula (13): 0.25z_{1,p,i} + 0.25 …. <= zi: Do you mean ‘=’ instead of ‘<=’? Otherwise this is redundant or?

**Author response:** In this constraint it is important to distinguish between global $z_{i}$ and local $z_{1/2,p/n,i}$. The latter register for which target region a cut of reaction i is required in order to disable that target. The global $z_{i}$basically is a logical OR over the local $z_{1/2,p/n,i}$ and thus shows that a cut is needed to disable at least one target. The global/local distinction is required to allow cut sets on the local target level that are independent of each other. The overall MCS is then the union of these local cut sets and represented by the global $z_{i}$. Note that the independence of the local cut sets is really necessary, because the overall MCS, when it is a superset of a local cut set for a given target, may not be a proper cut set for this target in the duality-based framework used here. The “=” would not work because not all z_{1/2,p/n,i} can be active at the same time. The “<=” ensures that z_i is one as soon as one z_{1/2,p/n,i} is one.

**Reviewer 1 comment 16:** I find the equations really hard to read because of how they are presented. Can you put the (in)equality signs all in the center, such that they are aligned?

**Author response:** We aligned the (in)equality signs as best as we could. But please note that the alignment will anyway be changed largely during production of the article.

**Reviewer 1 comment 17:**

Do you need z_{1,n,i} = 0 for irreversible reactions? Shouldn’t this be in Ar<=b (as claimed before)?

**Author response:** Actually, the variables z_{n,i} for irreversible reactions in eq. (9) and (likewise in eq. (13)) could indeed be left out completely and z_{i} then coincides with z_{p,i}. However, for the sake of readability (e.g., to use the term $z_{p,i}+ z_{n,i}=z_{i}$ for both reversible and irreversible reactions), we keep them but use the expression z_{n,i} = 0 for irreversible reactions to indicate that negative values of irreversible reactions in **v** do not count as a cut. Regarding the second question, the answer is NO, that is, irreversibility constraints of type r_i>=0 (if reaction i is irreversible) need not be included in A or T (or D) as they are separately treated in the MILP (however, inhomogeneous flux bounds such as r_i>=3 must be included). This was not clearly described in the previous version and we therefore revised this part (text around the equations (2)-(6)).

**Reviewer 1 comment 18:** What is the difference between constrained MCSs and the first extension? As far as I understood, cMCSs in <https://doi.org/10.1093/bioinformatics/btv217> can be also formulated for several desired and undesired phenotypes. However, not for those which interdict each other (e..g aerobic and anaerobic maximum growth). Please clarify.

**Author response:** Probably by mistake the reviewer cited a paper on regulatory MCS. The paper on constrained MCS (<https://doi.org/10.1016/j.ymben.2010.12.004>) from 2011 indeed already discussed the idea of taking into account multiple desired regions. However, it did not discuss multiple target regions and, most importantly, the calculation of MCS for multiple desired regions was so far only possible for the classical way of MCS calculation via elementary modes calculated in a preprocessing step, which is not applicable to genome-scale models. Hence, in the present study we now introduce for the first time the idea of multiple target regions AND allow the integration of multiple desired as well as of multiple target regions in the duality-based MCS calculation framework which was not possible before.

We now cite the paper in the first paragraph of the Extension 1 section and clarify what will be new in the Extension 1 presented in the present study.

**Reviewer 1 comment 19:** I would like to have more explanation on how to derive formula (9) (page 6). And it should be in a better readable format. What size is the identity matrix?

**Author response:** The duality-based approach (9) was introduced in the cited Reference of Ballerstein et al. (2012) and is based on the Farkas lemma. Reintroducing it here would inflate the Methods-section a lot and would mean to copy large parts from this paper. We think it is not necessary because the interested reader may follow the references given prior to formula (9). However, we extended the description of the different parts of the system shown in formula (9) and explain the matrix and the variables and their relationships with respect to the dual and the primal system in more detail (see also our response to comment 1 of reviewer 2). We also improved the alignments in eq. (9) and give now explicitly the dimension of the identity matrix in the text.

**Reviewer 1 comment 20:** Extension 3: DOI 10.1007/s10295-014-1576-3 add to references

**Author response:** Thanks, reference added

**Reviewer 1 comment 21:** page 9: MCS3 and MCS4 are part of the WT too, thus the additional reactions are not necessary here, or?

**Author response:** The wild type in the second row and first column is marked by a solid black line and does not contain the augmented solution space that result from the reaction addition candidates (the latter are only available for addition). The remaining solution space of MCS3 is indeed a subset of the WT and thus no reactions are added. In contrast, mutant MCS4 requires additional reactions for its solution space to intersect, as demanded, with desired region D1. This example was intentionally chosen to point out that the MCS algorithm delivers a set of solutions to a problem, some of which may or may not have reaction additions. However, both additions and deletions follow the principle of (cost-)minimality so that an MCS never contains unnecessary reaction additions (apart from the case of cost-free additions; see response to comment 7).

We edited the figure to make this more intuitive. We labeled the plane of “reaction addition candidates” and colored only the space in MCS4 that became accessible through the reaction additions.

**Reviewer 1 comment 22:** page 10, first sentence, typo: additionAL candidates

**Author response:** Corrected for clarity.

**Reviewer 1 comment 23:** page 10: Why can exchange reaction not be deleted? And if so, is this integrated in the MILPs? What if a reaction which is directly coupled to an exchange reaction is deleted?

**Author response:** A deletion of exchange reactions via genes is often not possible, for example, because transport may occur via diffusion (e.g. CO2 release) or because transporters are not known or not specific. In the example we therefore assumed the typical case that they are not targetable. Clearly, in general, if an exchange reaction IS associated with a targetable gene (e.g., glucose PTS in E. coli) then we can allow the deletion of the exchange reaction and treat it as all other reactions in the MILP. To clarify this issue, we now write for the example in Figure 1:

*“Dashed reaction arrows with yellow captions indicate exchange reactions (the latter are assumed to happen spontaneous, i.e., they are non-targetable).”*

**Reviewer 1 comment 24:** page 14: rule 2 and rule 3 can be merged. If a gene is essential for an essential reaction it should be marked as protected. Or am I missing something? Isn’t rule 2 contained in rule 3?

**Author response**: The reviewer describes GPR compression rule 3 that protects genes that are essential for essential reactions. Rule 2, instead, protects genes that only occur in essential reactions. Even though rules 2 and 3 are similar and often overlap, they are different and genes fulfilling rules 2 may not fulfill rule 3 (and vice versa):

A gene that fulfills rule 2 targets solely essential reactions. Note that it may itself not be essential since there might be two alternative genes for an essential reaction. So even though the gene might in principle be targetable, it cannot help in blocking the target region because it targets only reactions that will per definition remain unblocked in the final strain design. It can thus be marked as non-targetable as its knockout is never useful.

In contrast, a gene that fulfills rule 3 is definitely essential for an essential reaction and is thus per se non-targetable.

Rules 2 and 3 are thus different and require separate attention.

**Reviewer 1 comment 25:** table 1: EColiCore2, first column “No compression”, rows “# MCS found for compressed network” and “# MCS found after decompression”: Why 6025 and 6015? Shouldn’t it be the same?

**Author response:** Sorry, this was a typo and has been corrected.

**Reviewer 1 comment 26:** what is row “Reactions”? At the top, the number of reactions is 502 (core), resp 2715 (genome-scale), but the row contains higher numbers for the non-compressed network. Please explain. (Including the GPR rules delivers a smaller number too). Same for the number of species.

**Author response:** The row “Reaction” (now “# Reactions”) contains the number of reactions in the respective network. For the non-compressed network, the number of reactions and species is higher than in the original network due to the integration of GPR rules, which is done via introduction of pseudo-reactions and pseudo-species as explained in the Methods part.

**Reviewer 1 comment 27:** I am missing a legend which explains the rows and columns. It is also confusing that the rows say “compressed network”, even though the columns distinguish between compressed and     decompresses network too. Seems to be redundant and is confusing.

**Author response:** We agree that it was misleading to have “(compressed network)” in the row even though there are two columns with an uncompressed network. We now removed “(compressed network)” in the row identifier since it should be clear from the semantics what is meant. We also added a “#” in all row identifiers whenever a number is referenced.

In the legend of Table 1 we added these two sentences:

*“The rows “#Genes” and “#GPR rules” refer to the respective number of genes and GPR rules eventually integrated in the network after GPR rule compression (as long as the latter is conducted; otherwise it refers to the original number of genes and GPR rules). Likewise, the rows “#Species”, “#Reactions”, ”#Targetable reactions/genes” refer to the numbers of species, reactions, and targetable genes/reactions, respectively, after integration of the (compressed/non-compressed) GPR rules and/or after network compression.”*

**Reviewer 1 comment 28:** It is sometimes hard to distinguish between examples and actual constraints for the programs. Can the authors clarify this more? Maybe introducing an “example-environment”.

**Author response:** We tried to follow the suggestion of the reviewer and tested several variants to use a separate equation numbering for example equations. However, in the end, the PLOS style does not allow separated equation numberings (e.g. E1, E2 for example equations) and it is therefore hard to separate it. However, only a few example equations are numbered, namely if they are later referenced, hence, the majority of example equations can easily be recognized by the property that they have no number.

**Reviewer #2**:

The MCS methodology has been one of the major breakthroughs derived from the field of Systems Biology. The extensions of the MCS approach presented by Professor Klamt and colleagues are really interesting and valuable for different practical applications. In my opinion, this article deserves publication in Plos Computational Biology. However, I have some comments that, in my opinion, could improve the manuscript.

**Author response**: We thank the reviewer for the positive assessment of our manuscript.

**Reviewer 2 comment 1:** For readers not familiar with the MCS approach, the relationship between variables in the dual problem and constraints in the primal could be described in pages 5-6.

**Author response:** We agree with the reviewer and extended the description of system (9) to explain equations/variables of the dual and of the primal system. Below equation (9) we now write:

*“The blocking of the target region is addressed in the first two rows of the matrix in (9) via a duality-based relationship where the stoichiometric matrix* $\boldsymbol{N}$ *and the matrix* $\boldsymbol{T}$ *for the target region are used with their transposed version (cf. [21]). The associated variables for this dual subsystem are* $\boldsymbol{u}$*,* $\boldsymbol{v}$ *and* $\boldsymbol{w}$*. We note that there are variants in treating the* $n\times n$ *identity matrix* $\boldsymbol{I}$ *and its associated vector* $\boldsymbol{v}$ *(for example, reversible and irreversible reactions can be separated and the reversible be split into a positive and a negative part [18]) but we choose the above version, which is more concise. In contrast to the target region, the requirements for the desired region (third and fourth row in the matrix in eq. (9) as well as the flux bounds for the associated flux variable* $\boldsymbol{r}$*) are given in their primal representation directly expressing eq. (1), (2) and (6). The continuous dual (*$\boldsymbol{u,}\mathbf{v,w}$*) and primal (r) variables are interconnected via the binary* $z_{i}$ *variables. Each* $z_{i}$ *is linked with* $v_{i}$ *via two other binary variables (*$z_{p,i}$ *and* $z_{n,i}$*). Effectively, cuts are marked by* $z_{i}$ *that are equal to 1, which happens if a reversible reaction* $i$ *has a non-zero* $v_{i}$ *or if an irreversible reaction* $i$ *has a positive* $v_{i}$ *(hence, negative entries for irreversible reactions in* $\boldsymbol{v}$*do not count).”*

**Reviewer 2 comment 2:** The objective function for the first extension (multiple targets) could be defined after Eq. (13). By the way, this new approach is really elegant. However, the number of variables is double. In Table 2, you describe the computation time in the case of 2,3-BDO and ATP maintenance. Is it worthy in terms of computation time? Why not resolving 2632*2 (max/min ATP maintenance reaction) LPs to filter those satisfying ATP maintenance?

**Author response:** Regarding the objective function of the first extension (multiple targets/desired regions), we now mention directly after eq. (13) that the same objective function (10) (as used for the standard system) can be applied.

Yes, it is true that the number of variables with multiple target/desired systems increases quickly as the separate variables must be introduced for each region. The reviewer is also right that in the case of two desired regions (e.g., high 2,3-BDO synthesis + high ATP maintenance) one may first compute the MCS for high 2,3-BDO synthesis and afterwards check for the demanded minimum ATP maintenance rate (scenario 2 in table 2). However, this works only for the case when exclusively reaction knockouts (and no reaction additions) are allowed: with reaction additions, it may happen that all found MCS for the first desired constraint (high 2,3-BDO synthesis) violate the second desired constraint (high ATP synthesis), hence, all MCS would have to be discarded. Nevertheless, suitable MCS for both desired constraints may exist that require the addition of certain reactions. Hence, only if the second desired constraint would have been known during the calculation of the MCS, suitable MCS would be found. Furthermore, we normally observe that it is beneficial (in terms of computation time) to directly account for multiple desired regions in the MILP (instead of filtering out invalid MCS afterwards) as this reduces the space of feasible solutions and thus shortens the runtime. However, as the reviewer observed correctly, in the previous version of the manuscript the case with two desired regions (scenario 4) was slower than with one desired region (scenario 3). In the meantime we had further optimized the MILP setup in CPLEX by which the computation times in Tables 1 and 2 slightly changed. In fact, now we see that scenario 4 (two desired regions) is two times faster than scenario 3 (one desired region).

**Reviewer 2 comment 3:** Why the number of MCSs found after decompression in EcoliCore2 is different to the uncompressed case (6015 vs 6025)?

**Author response:** Thanks, this was a typo and has been corrected.

**Reviewer 2 comment 4:** I like a lot your approach to gene MCSs. It is clear the effect of GPR compression. However, it would be nice to compare your approach with the one presented in Apaolaza, 2019 in order to calculate synthetic lethals.

**Author response:** As suggested by the reviewer, we used our approach to compute synthetic lethals in iML1515 and compared the results and runtimes to those from the gMCS function introduced by Apaolaza. We computed all lethal gene-knockout combination in iML1515 up to the size of 4 and both approaches returned identical results thus showing consistency of both approaches. The comparison is mentioned in the results chapter and a script to repeat these computations was added to the GitHub repository.

We added the following paragraph to the results section:

*“Next we compared our approach for calculating MCS with integrated GPR associations with the gene cut set (gMCS) approach of Apaolaza et al. [26,27]. The latter determines in a preprocessing step a mapping of minimal gene knockout sets required to block at least one reaction. As the gMCS method can so far only deal with target reactions but not with desired regions we used the calculation of synthetic lethals (SL; combinations of reaction knockouts that block growth) in the genome-scale iML1515 network as benchmark. We enumerated all SL up to size 4 and found with both methods the identical set of 889 MCS which confirms their consistency. For the runtimes we determined 65 minutes with our methods and 163 minutes with gMCS. The scripts of these calculations are also available at the provided GitHub link.”*
